# Functional and structural outcomes in paediatric myelin oligodendrocyte glycoprotein antibody-associated disease (MOGAD): a prospective study

**DOI:** 10.1007/s10633-026-10082-1

**Published:** 2026-02-19

**Authors:** Flavia C. Gericke, James V. M. Hanson, Annette Hackenberg, Christina Gerth-Kahlert

**Affiliations:** 1 Department of Ophthalmology, University Hospital Zurich and University of Zurich, Zurich, Switzerland; 2 Department of Neuropediatrics, University Children’s Hospital Zurich, Zurich, Switzerland

**Keywords:** Myelin oligodendrocyte glycoprotein IgG, Visual evoked potential, Optic neuritis, Optical coherence tomography, Peripapillary retinal nerve fibre layer

## Abstract

**Purpose:**

Myelin oligodendrocyte glycoprotein antibody-associated disease (MOGAD) is a rare autoimmune disease affecting the central nervous system, frequently manifesting with optic neuritis (ON). Despite often favourable functional visual recovery, structural retinal changes may persist. This prospective study assessed the utility of functional and structural visual outcome measures in paediatric MOGAD.

**Methods:**

Children with serologically confirmed MOGAD were recruited from across Switzerland, alongside age-matched healthy controls. All participants underwent pattern-reversal visual evoked potentials (VEP; 15′ and 60′ check sizes), spectral-domain optical coherence tomography (OCT), high- and low-contrast distance visual acuity (VA), near VA, and colour vision testing. VEP outcomes were P100 peak times, and OCT outcomes were peripapillary retinal nerve fibre layer (pRNFL) thickness (global, temporal, nasal, and papillomacular bundle (PMB)), macular volume, and central retinal thickness. Discriminative performance was assessed using receiver operating characteristic (ROC) analysis.

**Results:**

Twelve paediatric MOGAD patients (10.9 ± 3.1 years) and twelve age-matched controls (11.5 ± 2.8 years) were included. VEP P100 peak times were generally comparable between patients and controls. pRNFL thickness appeared lower in patients than controls. ROC analysis showed excellent to outstanding discrimination for global, PMB, temporal, and nasal pRNFL sectors, both overall and in ON+ and ON– subgroups. VEP P100 peak times exhibited fair or poor discrimination overall, however 15′ check sizes showed excellent discrimination between ON+ and control eyes.

**Conclusions:**

In our cohort, OCT-derived pRNFL thickness differentiated MOGAD from controls with high accuracy, including in eyes without prior ON. VEPs showed limited utility, supporting OCT as a more sensitive marker of subclinical structural involvement.

## Introduction

Myelin oligodendrocyte glycoprotein antibody-associated disease (MOGAD) is a rare inflammatory autoimmune disorder of the central nervous system (CNS) encompassing a heterogeneous clinical spectrum [1–3]. Population-based studies estimate a prevalence of 1.3–2.5 per 100 000 and an annual incidence of 3.4–4.8 per million, with reported incidence rates of 3.1 per million in children and 1.6–2.4 per million in adults [4, 5]. Clinical presentations vary by age, with acute disseminated encephalomyelitis (ADEM) more commonly observed in younger children, and optic neuritis (ON) occurring more frequently in older children [6–10]. Although the disease course is typically monophasic, relapses may occur in a subset of patients. In children, both the relapse rate and cumulative disability burden, such as persistent visual, motor, or cognitive deficits following demyelinating episodes, are generally lower than in adults, with more favourable recovery profiles and reduced long-term structural damage [2, 11]. Early seroconversion to MOG-IgG negativity within the first year after disease onset has been associated with a decreased risk of future relapses [12, 13].

ON is a common manifestation in paediatric MOGAD and is typically characterised by acute, often severe unilateral or bilateral visual loss, which may be accompanied by optic disc oedema, periocular pain, and impaired colour perception [3, 14–16]. While visual acuity generally recovers well in children, optical coherence tomography (OCT) often reveals residual thinning of the peripapillary retinal nerve fibre layer (pRNFL), which can continue to progress for up to 12 months following an ON episode [7, 17]. Repeated ON episodes have been associated with cumulative retinal damage, whereas functional parameters, such as visual acuity, often recover irrespective of the number of relapses [7, 18, 19].

This discrepancy between functional recovery and persistent structural damage underscores the importance of assessing the utility of complementary visual, electrophysiological, and retinal measures in paediatric MOGAD. OCT offers quantitative structural markers, particularly through measurements of pRNFL thickness [14, 17, 20]. In parallel, visual evoked potentials (VEP) assess functional integrity along the visual pathway up to and including the visual cortex [21]. The VEP P100 peak time provides an objective index of conduction delay in inflammatory optic neuropathies and complements structural assessment when stimulus parameters (e.g., check size) are considered [22]. For example, pattern-reversal VEP is widely used to detect optic pathway involvement, including clinical and subclinical ON, in multiple sclerosis, and abnormalities may persist despite recovery of visual acuity [23]. Longitudinal VEP follow-up in children with ON has similarly demonstrated substantial clinical and electrophysiological improvement within the first year after the acute episode, with VEP abnormalities normalising in a subset of eyes but persisting in others [24]. In MOGAD, VEP peak times are often mildly prolonged, reflecting demyelination, although paediatric data remain sparse [7]. Additionally, high- and low-contrast visual acuity (HCVA, LCVA) and colour vision testing may offer further clinical information, especially when structural and electrophysiological findings diverge. At the present time, it remains unclear which of these functional and structural outcomes have the greatest utility in the diagnosis and investigation of paediatric MOGAD. We aimed to investigate this in detail in a prospective study.

## Methods

### Study population

Children with serologically confirmed MOGAD, initially diagnosed between 2 and 14 years of age, were recruited from throughout Switzerland, along with locally recruited age-matched control subjects. In the MOGAD group, individual eyes of patients were further stratified into subgroups with and without a history of optic neuritis (ON+ and ON–, respectively). Classification was based on retrospective review of electronic medical records. Clinical examinations were conducted between January 2024 and March 2025 at the Department of Ophthalmology, University Hospital Zurich. Patients were recruited in collaboration with the Department of Neuropediatrics at the University Children’s Hospital Zurich and the Swiss Paediatric Inflammatory Brain Disease Registry (Swiss-Ped-IBrainD) of the University of Bern.

Inclusion criteria for the MOGAD group were MOG-IgG seropositivity, a previous acute presentation of a clinical phenotype associated with MOGAD, and provision of written informed consent. Eyes were excluded if an episode of ON had occurred within the six months preceding examination, to minimise confounding of pRNFL thickness measurements by acute inflammatory changes [25].

Control participants were eligible if, according to parental report and existing clinical documentation (e.g., paediatric check-ups), they had normal ocular status, good general health, no history of ocular or systemic disease, and no high refractive error (defined as spherical equivalent >  ± 6.0 dioptres or astigmatism > 2.0 dioptres).

### Visual evoked potential

Pattern-reversal VEPs were recorded in accordance with the contemporary published standard of the International Society for Clinical Electrophysiology of Vision (ISCEV) [22] on an Espion system (Diagnosys LLC, Lowell MA, U.S.A.). VEPs were elicited using 15′ and 60′ black-and-white checkerboard stimuli presented on a cathode ray tube monitor at a distance of 1 m and reversing in polarity at 2.05 reversals per second (equivalent to just over 1 Hz), with the recording electrode at O^Z^, reference at F^Z^, and ground at the vertex (C^Z^). Field size was 21° horizontally and 16° vertically, and the black and white checks were presented with a mean luminance of 58 cd · m^−2^ and Michaelson contrast of 98%. A minimum of 50 sweeps were averaged for each measurement, with two measurements per eye and per check size being performed to verify reproducibility. The primary outcome measure was the P100 peak time in milliseconds (ms), averaged from both measurements per eye and condition.

### Optical coherence tomography

Retinal imaging was performed using spectral-domain optical coherence tomography (SD-OCT; SPECTRALIS, Heidelberg Engineering, Heidelberg, Germany). Image quality was assessed in accordance with the OSCAR-IB criteria [26, 27]. For each participant, pRNFL ring scans (diameter 12°, High-Resolution (HR) mode, 100 Automatic Real-Time Tracking (ART) images averaged) were acquired, permitting average thickness quantification of the global, temporal, nasal, and papillomacular bundle (PMB) sectors. In addition, macular volume scans (30° × 25° pattern composed of 31 scans separated by 245 µm, HR mode, ≥ 15 ART images averaged) were obtained to assess total macular volume and central retinal thickness (CRT). The inner limiting membrane, RNFL/ganglion cell layer border (pRNFL scans only), and Bruch’s membrane (macular volume scans only) were identified by the manufacturer's software (EyeExplorer V2.6.4; Heidelberg Engineering) and manually reviewed and corrected by a single investigator (FCG) when necessary.

### Visual acuity and colour vision

Monocular visual acuity (VA) for both distance and near vision was assessed in all participants. Distance VA was measured using Early Treatment Diabetic Retinopathy Study (ETDRS) and Sloan charts at high (100%) and low (2.5%) contrast, respectively, and was recorded as the logarithm of the minimum angle of resolution (logMAR), with each line of five letters corresponding to 0.10 logMAR and each individual letter to 0.02 logMAR. Near VA was assessed monocularly using a standardised Snellen near vision chart (C chart) with line-based presentation, using crowded optotypes (multiple C-rings per row). Snellen near VA values were converted to logMAR for easier comparison with HCVA and LCVA. All assessments of VA were performed with participants’ habitual optical correction.

Colour vision was assessed monocularly using the Mollon-Reffin Minimalist Colour Vision Test, a pseudoisochromatic test designed to evaluate red-green (Protan (P) and Deutan (D)) and blue-yellow (Tritan (T)) colour discrimination. In each trial, participants were asked to identify one coloured cap among five achromatic distractors of varying lightness. The test assesses six saturation levels along each colour axis and is suitable for use in children, including those as young as 3 years [28].

### Statistical analysis

VEP, OCT, VA, and colour vision data were descriptively summarised in three comparisons: all MOGAD patients vs age-matched controls, MOGAD ON+ vs age-matched controls, and MOGAD ON– vs age-matched controls. For OCT analyses, normative data for paediatric eyes were derived from the age-matched healthy control cohort described in Sect. "Study population". For continuous variables, medians and interquartile ranges were reported; for categorical variables, frequencies and proportions were used. Given the small sample size (dictated by the rarity of paediatric MOGAD) and exploratory nature of the study, no inferential statistical testing was performed. Instead, we focused on descriptive data analysis and receiver operating characteristic (ROC) curve analysis to evaluate the diagnostic performance of individual outcome measures. ROC analysis was used to determine the area under the curve (AUC) for VEP, OCT, and VA parameters in distinguishing MOGAD subgroups from control subjects, and was conducted according to the method described by Hanley and McNeil [29]. Interpretation of AUC values was based on the classification proposed by Hosmer and Lemeshow [30]. All data were analysed, and figures were generated using Prism 10 (GraphPad Software Inc., La Jolla, CA, USA).

### Ethical approval and consent

The study adhered to the tenets of the Declaration of Helsinki. Ethical approval for the study was obtained from the Cantonal Ethics Committee of Zurich, Switzerland (BASEC-ID: 2022–01562). Written informed consent for participation, data use, and publication was provided by the parents of all patients and control subjects.

## Results

### Cohort description

Twelve paediatric patients with serologically confirmed MOGAD were examined (mean age 10.9 ± 3.1 years; median 10.3 (Q1–Q3 8.2–13.1); female (F): male (M) ratio 5:7), alongside twelve age-matched healthy controls (mean age 11.5 ± 2.8 years; median 11.0 (9.3–13.0); F:M ratio 11:1). Of the MOGAD cohort, 8/12 patients had previously experienced ON, including five with bilateral (two of whom had sequential ON) and three with unilateral ON, yielding a total of 13/23 ON+ affected eyes. One eye in the MOGAD cohort was excluded due to anisometropic amblyopia. Detailed clinical and demographic characteristics are summarised in Table 1.

**Table 1:** Demographic and clinical characteristics of the MOGAD and healthy control groups

MOGAD	
N (eyes)	12 (23*)
**Mean age, years (SD)**	10.9 (3.1)
**Sex (F/M)**	5/7 (42%/58%)
*Diagnosis*	
ON	2 (17%)
ADEM-ON	4 (33%)
ADEM-ON-TM	1 (8%)
ADEM-TM	1 (8%)
ADEM	3 (25%)
NMOSD-ON	1 (8%)
*History of ON presentation*	
Unilateral	3 (38%)
Bilateral	5 (62%)
**ON+ eyes**	13 (57%)
**ON– eyes**	10 (43%)
*Control*	
N (eyes)	12 (24)
Mean age, years (SD)	11.5 (2.8)
Sex (F/M)	11/1 (92%/8%)

Overview of mean age, sex distribution, clinical diagnoses, and ON history in paediatric MOGAD patients and age-matched healthy controls. Percentages for ON presentation were calculated among ON + patients (n = 8); percentages for ON + and ON − eyes were calculated among all MOGAD eyes (n = 23). One eye was excluded from the MOGAD group due to amblyopia (*). *Abbreviations: ADEM, acute disseminated encephalomyelitis; ADEM-ON, ADEM with optic neuritis; ADEM-ON-TM, ADEM with optic neuritis and transverse myelitis; ADEM-TM, ADEM with transverse myelitis; MOGAD, myelin oligodendrocyte glycoprotein antibody-associated disease; NMOSD-ON, neuromyelitis optica spectrum disorder with ON; ON, optic neuritis; ON*+ *eyes, MOGAD eyes with a history of ON; ON− eyes, MOGAD eyes without a history of ON; F, female; M, male; N, number; SD, standard deviation*

**Fig. 1:**
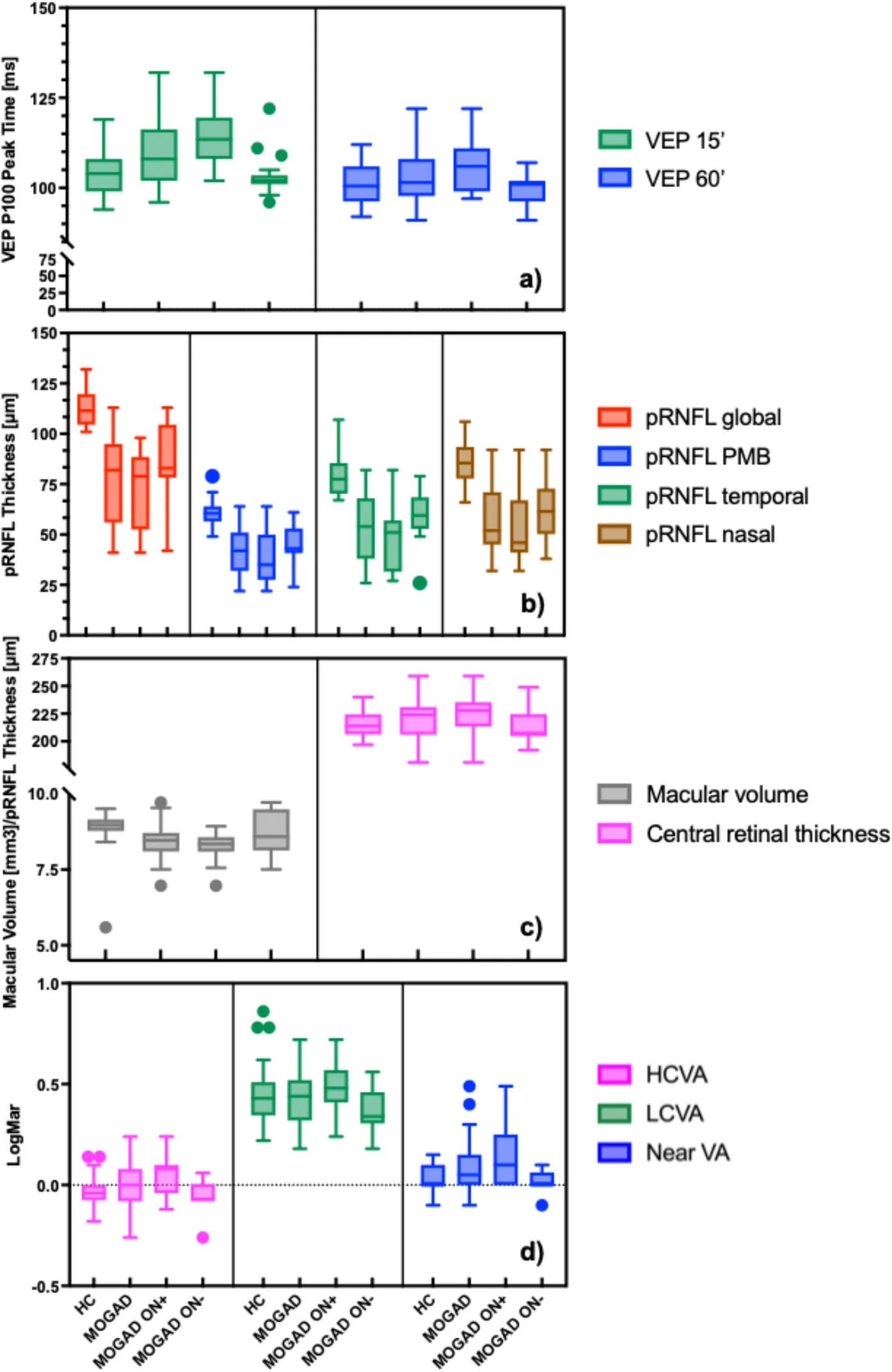
(**a**–**d**): Comparison of functional, structural, and visual acuity measures across MOGAD subgroups and healthy controls Tukey-style box-and-whisker plots illustrate group differences in (a) VEP P100 peak time, (b) pRNFL thickness (global, PMB, temporal, nasal), (c) macular volume and CRT and (d) visual acuity measures (logMAR) between HC, all MOGAD eyes, and MOGAD subgroups with (ON+) and without (ON–) prior ON. The x-axis labelling (HC, MOGAD, MOGAD ON+ , MOGAD ON–), shown explicitly in (d), applies to all panels (a–c). *CRT, central retinal thickness; HC, healthy controls; logMAR, logarithm of the minimum angle of resolution; MOGAD, myelin oligodendrocyte glycoprotein antibody–associated disease; OCT, optical coherence tomography; ON*+ *, MOGAD eyes with a history of optic neuritis; ON–, MOGAD eyes without a history of optic neuritis; PMB, papillomacular bundle; pRNFL, peripapillary retinal nerve fibre layer; VA, visual acuity; VEP, visual evoked potential.*

For functional measures, VEP P100 peak times were slightly prolonged in ON+ eyes compared with controls, most evident with 15′ stimulation (113.5 ms (108.0–119.5) vs 104.0 ms (99.0–108.0)) (Table 2, Fig. 1a). At 60′, ON + eyes showed a smaller difference (106.0 ms (99.0–111.0) vs 100.5 ms (96.3–106.0)). ON– eyes were comparable to controls at both check sizes (15′: 102.0 ms (101.0–103.5) vs 104.0 ms (99.0–108.0); 60′: 101.0 ms (96.3–102.0) vs 100.5 ms (96.3–106.0)). Across all MOGAD eyes, median peak times (15′: 108.0 ms, 60′: 101.5 ms) overlapped with control values.

**Table 2:** Median and interquartile range (Q1–Q3) of functional and structural visual outcomes in MOGAD patients and healthy controls

Median (Q1–Q3)	MOGAD			Control
	(n = 23)	ON+ (n = 13)	ON– (n = 10)	(n = 24)
VEP 15′	108.0 (102.0 − 116.3)	113.5 (108.0 − 119.5)	102.0 (101.0 − 103.5)	104.0 (99.0 − 108.0)
VEP 60′	101.5 (97.8 − 108.0)	106.0 (99.0 − 111.0)	101.0 (96.3 − 102.0)	100.5 (96.3 − 106.0)
pRNFL global	82.0 (56.0 − 95.0)	79.0 (52.5 − 88.5)	83.0 (78.3 − 104.5)	111.5 (104.5 − 119.8)
pRNFL PMB	42.0 (32.0 − 51.0)	35.0 (27.5 − 50.0)	43.0 (40.8 − 53.0)	60.5 (56.5 − 64.0)
pRNFL temporal	54.0 (38.0 − 68.0)	51.0 (31.5 − 57.0)	59.5 (52.8 − 68.5)	77.5 (70.3 − 85.5)
pRNFL nasal	52.0 (45.0 − 71.0)	46.0 (41.0 − 67.0)	61.5 (50.3 − 72.8)	85.5 (77.8 − 93.5)
Macular volume	8.5 (8.1 − 8.7)	8.4 (8.1 − 8.6)	8.6 (8.1 − 9.5)	9.0 (8.8 − 9.2)
CRT	224.0 (206.0 − 231.0)	228.0 (213.5 − 235.5)	207.5 (204.8 − 224.8)	214.0 (206.3 − 224.5)
HCVA	0.00 (-0.08 − 0.08)	0.08 (-0.04 − 0.10)	-0.07 (-0.08 − 0.01)	-0.04 (-0.08 − 0.00)
LCVA	0.44 (0.32 − 0.52)	0.48 (0.41 − 0.57)	0.34 (0.31 − 0.46)	0.43 (0.35 − 0.51)
Near VA	0.05 (0.00 − 0.15)	0.10 (0.00 − 0.25)	0.00 (0.00 − 0.06)	0.00 (0.00 − 0.10)

Data are shown for all MOGAD eyes vs controls, as well as stratified comparisons of MOGAD eyes with (ON +) and without (ON–) a history of optic neuritis, each matched to age-similar controls. VEP and OCT values are rounded to one decimal place; visual acuity values are rounded to two decimal places to reflect the resolution of the logMAR scale. *Abbreviations:CRT, central retinal thickness (µm); HCVA, high-contrast visual acuity at 100% contrast (logMAR); LCVA, low-contrast visual acuity at 2.5% contrast (logMAR); MOGAD, myelin oligodendrocyte glycoprotein antibody-associated disease; Near VA, near visual acuity (logMAR); ON*+ *, MOGAD eyes with a history of optic neuritis; ON− , MOGAD eyes without a history of optic neuritis; PMB, papillomacular bundle; pRNFL, peripapillary retinal nerve fibre layer thickness (µm); VA, visual acuity; VEP 15′, visual evoked potential P100 peak time with 15′ check size (ms); VEP 60′, visual evoked potential P100 peak time with 60′ check size (ms)*

**Fig. 2:**
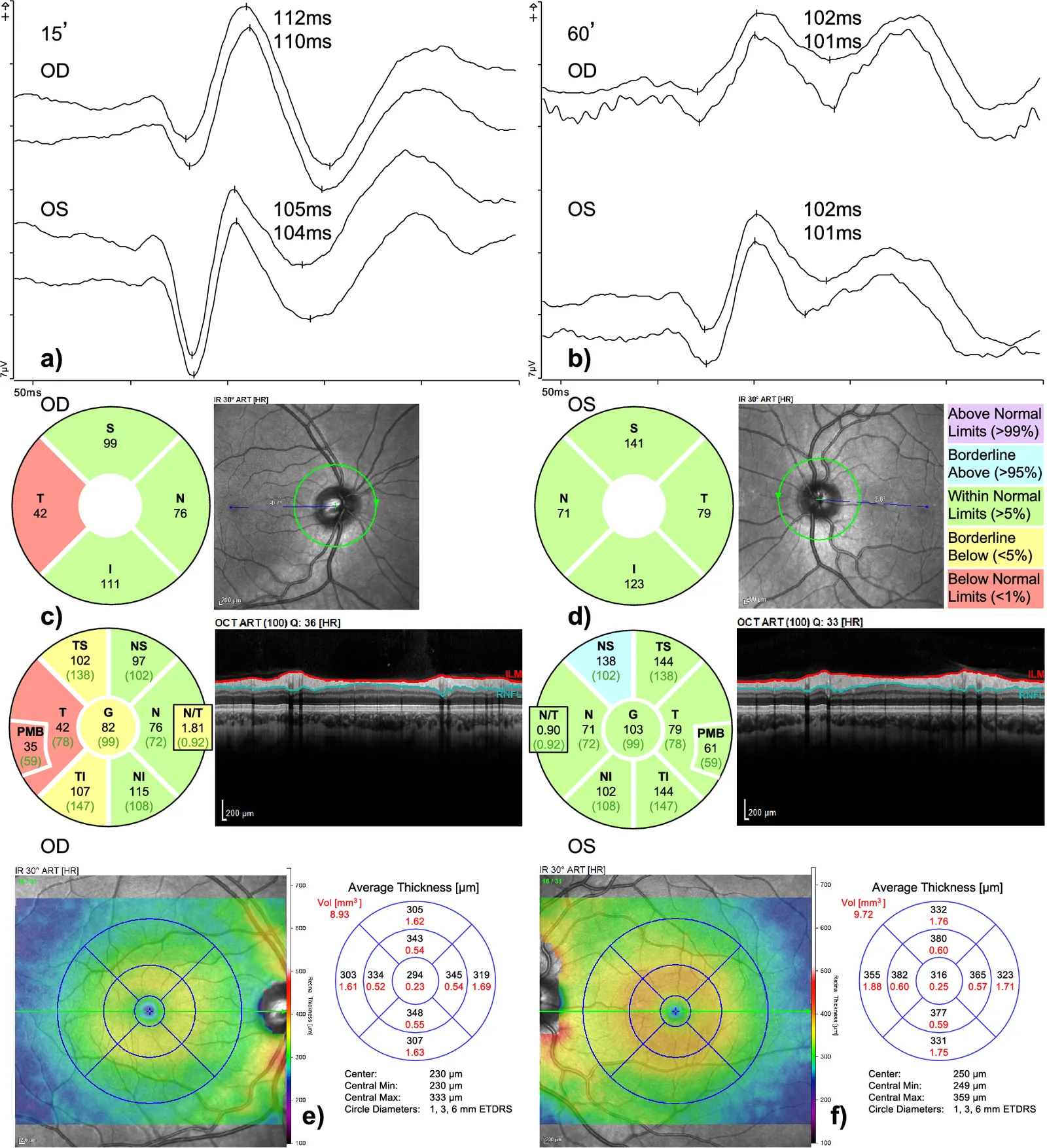
Representative paediatric MOGAD ON+ case with previous optic neuritis in the right eye Overview of structural and functional results in a paediatric MOGAD patient with right-sided ON, showing (a, b) pattern-reversal VEP P100 peak times for 15′ and 60′ stimulation, (c, d) pRNFL thickness sectors, and (e, f) macular volume and CRT. VEP P100 peak times are mildly prolonged for the 15′ stimulation in the affected eye (110/112 ms vs 104/105 ms), whereas 60′ responses are identical. OCT shows thinning of the pRNFL in the ON+ right eye, most pronounced in the PMB and temporal sectors (PMB 35 vs 61 µm; temporal 42 vs 79 µm), along with lower macular volume and CRT (8.93 vs 9.72 mm^3^; 230 vs 249 µm). Distance and near visual acuity (logMAR) were 0.02/0.48/0.00 (HCVA/LCVA/near VA) in the ON+ right eye and − 0.26/0.18/− 0.10 in the fellow eye, indicating reduced LCVA in the affected eye, while HCVA, near VA and colour vision were normal bilaterally (P1, D1, T1). Post-processing of some individual panels was performed to optimise image clarity, however no changes to individual numbers or letters, or colour processing, was performed. *CRT, central retinal thickness; D, Deutan; HCVA, high-contrast visual acuity; LCVA, low-contrast visual acuity; MOGAD, myelin oligodendrocyte glycoprotein antibody-associated disease; OD, right eye; ON, optic neuritis; ON*+ *, eye with a history of optic neuritis; OS, left eye; P, Protan; PMB, papillomacular bundle; pRNFL, peripapillary retinal nerve fibre layer; T, Tritan; VA, visual acuity; VEP, visual evoked potential.*

Structurally, peripapillary RNFL thickness was consistently reduced in MOGAD eyes compared with controls across all sectors. Median global pRNFL was 82.0 µm (56.0–95.0) in MOGAD vs 111.5 µm (104.5–119.8) in controls (− 26.5% percentage difference relative to controls), with more pronounced thinning in ON+ eyes (vs controls, − 29.1%) and in the PMB and nasal sectors (− 31.0% and − 39.2%, respectively). Reduced pRNFL was likewise observed in ON− eyes relative to controls (global: 83.0 µm (78.3–104.5); − 25.6%). Macular volume was slightly lower in MOGAD (8.5 mm^3^ (8.1–8.7)) than in controls (9.0 mm^3^ (8.8–9.2)), while CRT showed substantial overlap between groups. Visual acuity was largely preserved across groups (Table 2, Fig. 1d). Colour vision was normal in all but one ON+ eye. Figure 2 shows a representative MOGAD ON+ case with a history of right-sided ON.

### ROC analysis

Diagnostic performance was classified according to AUC thresholds as proposed by Hosmer and Lemeshow: ≥ 0.90 = outstanding, 0.80–0.89 = excellent, 0.70–0.79 = acceptable, 0.60–0.69 = fair, and < 0.60 = poor [30].

**Fig. 3:**
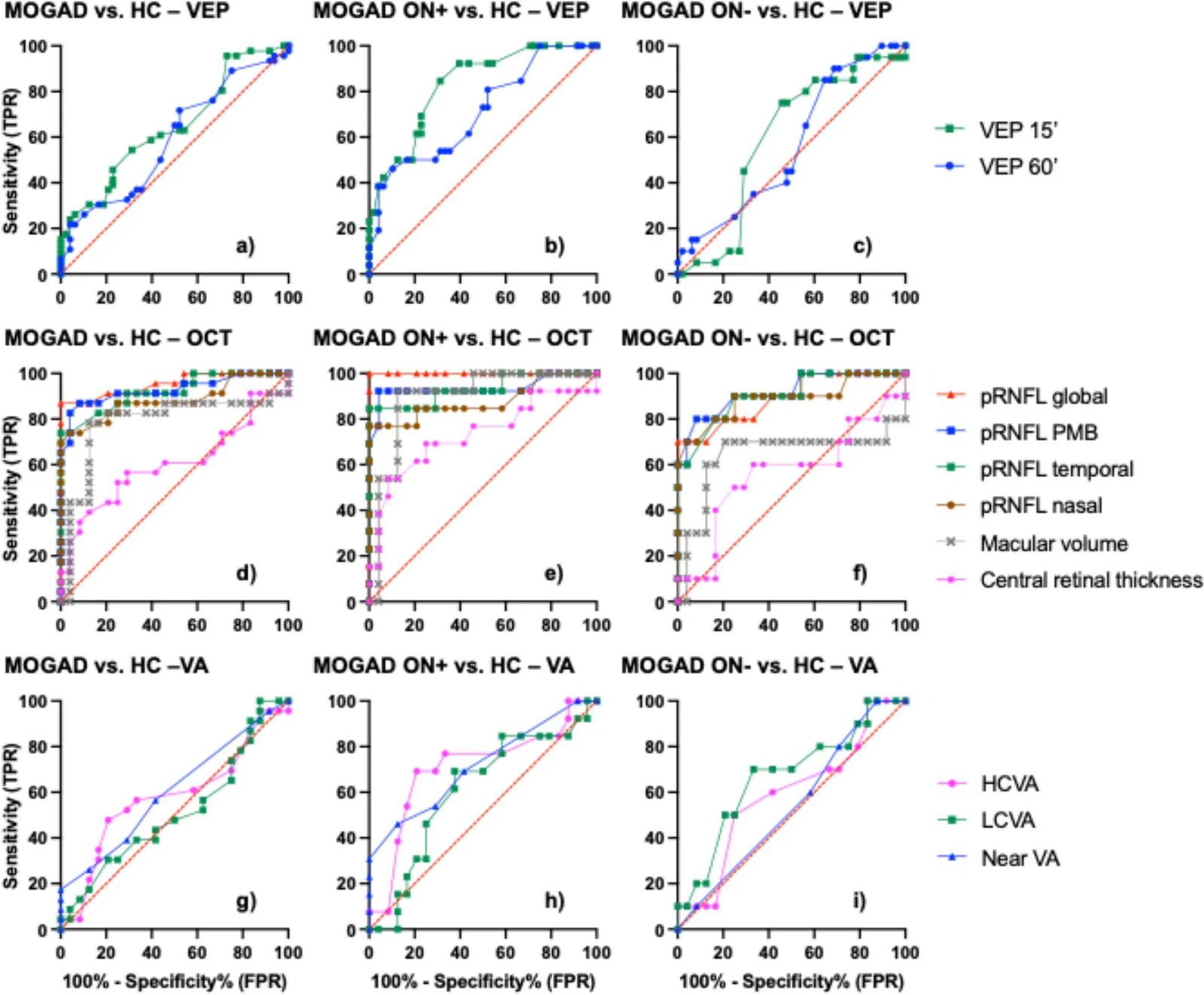
(**a**–**i**): ROC curve analysis of VEP, OCT, and visual acuity parameters in MOGAD subgroups and healthy controls Receiver operating characteristic (ROC) curves illustrating the discriminative ability of (a–c) visual evoked potentials, (d-f) OCT-derived parameters, and (g–i) visual acuity measures to distinguish MOGAD eyes from HC. Panels display data from all MOGAD eyes (a, d, g), MOGAD eyes with a history of optic neuritis (ON+) (b, e, h), and MOGAD eyes without prior ON (ON–) (c, f, i). The y-axis represents sensitivity (true positive rate), and the x-axis shows the false positive rate (100%–specificity). These axis labels apply to all panels (a–i). *FPR, false positive rate; HC, healthy controls; HCVA, high-contrast visual acuity; LCVA, low-contrast visual acuity; MOGAD, myelin oligodendrocyte glycoprotein antibody–associated disease; OCT, optical coherence tomography; ON*+ *, MOGAD eyes with a history of optic neuritis; ON–, MOGAD eyes without a history of optic neuritis; PMB, papillomacular bundle; pRNFL, peripapillary retinal nerve fibre layer; ROC, receiver operating characteristic; VA, visual acuity; VEP, visual evoked potential.*

Among functional parameters, VEP peak times showed variable performance (Fig. 3a-c, Table 3; AUCs and 95% confidence intervals (CI) were rounded to two decimal places). In ON+ eyes, 15′ stimulation reached the excellent range (AUC = 0.83, 95% CI 0.74–0.92), while 60′ showed acceptable discrimination (0.71, 0.59–0.83). In ON– eyes, AUC values were poor at both check sizes (15′: 0.59, 0.45–0.73; 60′: 0.56, 0.42–0.71), and in the full cohort discrimination was similarly low (15′: 0.65, 0.53–0.76; 60′: 0.59, 0.48–0.71).

**Table 3:** Area under the curve (AUC) values and 95% confidence intervals (CI) from ROC analysis comparing MOGAD eyes to healthy controls

ROC Analysis	MOGAD vs HC		MOGAD ON+ vs HC		MOGAD ON– vs HC	
	AUC	CI	AUC	CI	AUC	CI
VEP 15′	0.65	0.53–0.76	0.83**	0.74–0.92	0.59	0.45–0.73
VEP 60′	0.59	0.48–0.71	0.71	0.59–0.83	0.56	0.42–0.71
pRNFL global	0.95*	0.89–1.00	1.00*	1.00–1.00	0.89**	0.76–1.00
pRNFL PMB	0.93*	0.85–1.00	0.94*	0.83–1.00	0.92*	0.81–1.00
pRNFL temporal	0.92*	0.84–1.00	0.93*	0.84–1.00	0.90*	0.79–1.00
pRNFL nasal	0.88**	0.78–0.99	0.88**	0.74–1.00	0.89**	0.74–1.00
Macular volume	0.78	0.63–0.93	0.89**	0.78–1.00	0.64	0.38–0.90
CRT	0.60	0.44–0.77	0.73	0.54–0.92	0.56	0.33–0.80
HCVA	0.58	0.41–0.75	0.71	0.52–0.90	0.59	0.37–0.80
LCVA	0.51	0.34–0.68	0.61	0.42–0.80	0.67	0.46–0.87
Near VA	0.60	0.44–0.77	0.71	0.53–0.90	0.54	0.34–0.75

The table summarises diagnostic performance (AUC and CI) for functional and structural visual outcome measures across three comparisons: all MOGAD eyes vs controls, MOGAD ON+ eyes (with a history of optic neuritis) vs controls, and MOGAD ON– eyes (without a history of optic neuritis) vs controls. AUC values are rounded to two decimal places. Diagnostic performance classified as “outstanding” (AUC ≥ 0.90, marked *) or “excellent” (AUC 0.80–0.89, marked **) according to AUC thresholds as proposed by Hosmer and Lemeshow. *Abbreviations:AUC, area under the curve (receiver operating characteristic); CI, 95% confidence interval; CRT, central retinal thickness (µm); HC, healthy controls; HCVA, high-contrast visual acuity at 100% contrast (logMAR); LCVA, low-contrast visual acuity at 2.5% contrast (logMAR); MOGAD, myelin oligodendrocyte glycoprotein antibody-associated disease; Near VA, near visual acuity (logMAR); ON*+ *, MOGAD eyes with a history of optic neuritis; ON− , MOGAD eyes without a history of optic neuritis; PMB, papillomacular bundle; pRNFL, peripapillary retinal nerve fibre layer thickness (µm); VA, visual acuity; VEP 15′, P100 peak time with 15′ check size (ms); VEP 60′, P100 peak time with 60′ check size (ms)*

Structural OCT parameters showed the strongest overall performance across subgroups. In the full MOGAD cohort, global pRNFL achieved outstanding discrimination (0.95, 0.89–1.00), with PMB (0.93, 0.85–1.00), temporal (0.92, 0.84–1.00), and nasal (0.88, 0.78–0.99) sectors also performing in the excellent-to-outstanding range. Similar or higher values were observed in ON+ eyes, where global pRNFL yielded AUC = 1.00 (1.00–1.00) in this cohort. In ON– eyes, PMB and temporal pRNFL remained ≥ 0.90 despite the absence of clinically apparent ON. Nasal pRNFL, although showing the largest relative thinning (see 3.1), yielded slightly lower AUCs across all groups. Macular volume showed acceptable-to-excellent performance in the full cohort (0.78, 0.63–0.93) and ON+ eyes (0.89, 0.78–1.00), but only fair in ON– eyes (0.64, 0.38–0.90). CRT ranged from acceptable (0.73, 0.54–0.92, ON+) to poor (0.56, 0.33–0.80, ON–).

Visual acuity measures showed only fair or poor discrimination across all groups (Table 3).

To further assess discriminative accuracy, optimal cut-off values for pRNFL parameters were determined using the Youden Index (YI), as only these consistently demonstrated excellent or outstanding ROC performance across all groups. In the overall MOGAD cohort, a global pRNFL threshold of < 99.5 µm yielded 87% sensitivity, 100% specificity, and a Youden Index of 0.87. In ON+ eyes, the strongest performance was observed for PMB pRNFL < 51.5 µm with 92% sensitivity, 96% specificity, and YI = 0.88. In ON– eyes, global pRNFL < 99.5 µm achieved 70% sensitivity, 100% specificity, and YI = 0.70. Although a threshold was also calculated for global pRNFL in ON+ eyes (AUC = 1.00), only the PMB value is reported here to provide one representative cut-off per subgroup. A full overview of all thresholds and corresponding performance metrics is provided in Table 4.

**Table 4:** Threshold, sensitivity, specificity, and Youden Index for OCT-derived pRNFL parameters

Group	Parameter	Threshold (µm)	Sensitivity (%)	Specificity (%)	Youden Index
MOGAD vs HC	pRNFL global	99.5	87	100	0.87
	pRNFL PMB	52.5	87	92	0.79
	pRNFL temporal	63.5	74	100	0.74
	pRNFL nasal	67.0	74	96	0.70
MOGAD ON+ vs HC	pRNFL global	99.5	100	100	1.00
	pRNFL PMB	51.5	92	96	0.88
	pRNFL temporal	63.0	85	100	0.85
	pRNFL nasal	62.0	77	100	0.77
MOGAD ON– vs HC	pRNFL global	99.5	70	100	0.70
	pRNFL PMB	52.5	80	92	0.72
	pRNFL temporal	70.5	90	75	0.65
	pRNFL nasal	67.0	70	96	0.66

This table presents threshold values, sensitivity, specificity, and Youden Index for OCT-derived pRNFL parameters, comparing MOGAD subgroups with healthy controls. Sensitivity and specificity are rounded to the nearest whole number for clarity. Rule: classify as MOGAD if pRNFL < threshold. *Abbreviations: HC, healthy controls; MOGAD, myelin oligodendrocyte glycoprotein antibody-associated disease; ON*+ *, MOGAD eyes with a history of optic neuritis; ON− , MOGAD eyes without a history of optic neuritis; OCT, optical coherence tomography; PMB, papillomacular bundle; pRNFL, peripapillary retinal nerve fibre layer thickness (µm)*

## Discussion

This study aimed to characterise functional and structural alterations in paediatric MOGAD, with a focus on differentiating eyes with and without prior optic neuritis. OCT-derived parameters, especially pRNFL thickness in the global, temporal, and PMB sectors, showed strong discriminatory capacity between MOGAD eyes and healthy controls. Global pRNFL thickness yielded the highest AUC in the full cohort (0.95), with ON+ eyes reaching complete separation from controls (AUC = 1.00), although this may in part reflect the limited sample size in this subgroup. Notably, reduced pRNFL values were observed in ON– eyes, which also exhibited high AUCs in the temporal sectors, suggesting that OCT may detect subtle axonal loss even in the absence of overt inflammation. In contrast, macular volume and central retinal thickness showed lower AUCs and less consistent group separation; however, because we did not perform macular layer segmentation, we cannot rule out that segmented macular metrics, particularly GCIPL thickness, would perform similarly or better, as reported in cohorts using macular segmentation [27, 31].

Functional measures, including HCVA, LCVA and colour vision, were largely preserved in our cohort, consistent with the known dissociation between structural damage and visual performance in paediatric MOGAD [7, 8, 32]. While P100 peak times were mildly prolonged, particularly in ON+ eyes, the overall accuracy of VEPs remained inferior to that of OCT. In ROC analysis, 15′ stimulation in ON+ eyes achieved excellent discrimination, whereas 60′ performance was lower, and results in ON– eyes and the full cohort were poor. These findings would indicate that, in our cohort, peak time abnormalities are mainly confined to clinically affected eyes. Additionally, as discrimination performance in ON+ eyes appeared superior with 15’check sizes, it remains feasible that using smaller, non-standard check sizes may further improve performance. A recent study by Yano et al., based on an adult MOGAD cohort, found that all eyes with acute ON exhibited prolonged P100 peak times, whereas only 23% of eyes without acute ON, including both ON+ and ON– eyes, showed abnormal VEPs, with median peak time not significantly different from controls. In our paediatric cohort, ON– eyes (strictly defined as never affected by ON clinically or by history) consistently showed normal VEP peak times, further supporting that subclinical functional involvement is rare in MOGAD without clinical ON [33]. This may support findings that, in children, visual pathway conduction may recover despite persistent axonal loss owing to greater neuroplasticity [7, 16].

The observation of structural abnormalities in ON– eyes, particularly in the PMB and temporal sectors, raises the possibility of subclinical optic nerve involvement in paediatric MOGAD. Subclinical pRNFL thinning in the absence of ON is well documented in MS and is considered a marker of diffuse neuroaxonal degeneration [34, 35]. Wicki et al. observed bilateral retinal changes in MS patients following a first unilateral ON episode, suggesting that structural alterations may extend beyond the clinically affected eye [36]. In contrast, evidence for subclinical thinning in AQP4-IgG + NMOSD is more limited and heterogeneous across studies [27, 37, 38].

In MOGAD, Narayan et al. reported pRNFL loss in ON– eyes, suggestive of subclinical ON [10], and Vabanesi et al. described delayed VEP peak times despite preserved pRNFL thickness, indicating potential functional involvement without detectable structural damage [39]. In contrast, a multicentre longitudinal OCT study by Oertel et al. found no evidence for relapse-independent retinal neuroaxonal degeneration in MOGAD, suggesting that structural changes are predominantly relapse-driven [27]. In addition, Yano et al. found that subclinical VEP abnormalities in MOGAD were rare (23%) among eyes without acute ON, as previously defined, supporting a lower degree of subclinical optic nerve involvement compared with MS and NMOSD [23, 33]. Thus, MOGAD appears dominated by relapse-related inflammatory events with frequent lesion resolution, while chronic active lesions and persistent subclinical inflammation in MS drive progressive accumulation of silent lesions and neuroaxonal loss [40]. In line with these observations, longitudinal MRI studies have shown that new clinically silent brain and spinal cord lesions during remission are rare in MOGAD (reported in < 5% of follow-up scans in adults and 11–14% in children)[40], whereas asymptomatic lesion accrual is common in MS and contributes to long-term disease progression[40–42]. Notably, all of these studies were conducted in adult populations, and specific paediatric data remain scarce.

In this context, OCT may serve as a sensitive imaging modality to detect early structural involvement in paediatric MOGAD. While causality cannot be inferred from cross-sectional data, the differences observed in ON– eyes highlight the need for further investigation. Larger, longitudinal studies are required to determine the prevalence, underlying mechanisms, and clinical implications of ON– independent retinal alterations in this population.

Our study has limitations. Due to the rarity of paediatric MOGAD, the sample size was small, and the study was not statistically powered for formal between-group comparisons. Nevertheless, ROC analysis allowed the assessment of the discriminative performance of individual functional and structural measures, even in the absence of formal group comparisons [43]. The cross-sectional design precluded longitudinal assessment. Potential within-subject correlation in paired-eye data was considered a minor concern in the context of ROC analysis; however, possible future studies may benefit from using bootstrap-based confidence intervals or generalised linear mixed models to account for intra-subject clustering. Finally, our calculated pRNFL thickness cut-off values for optimal discrimination of paediatric MOGAD patients from healthy children of comparable age are specific to the Spectralis OCT device, although they are likely to be broadly comparable to devices from other manufacturers [44]. Pattern-reversal VEPs were recorded from a single midline occipital electrode (Oz) in accordance with the ISCEV standard, which precludes localisation of dysfunction to the pre- or post-chiasmal visual pathway [45]. Despite the rarity of post-chiasmal and isolated chiasmal lesions in MOGAD-ON [3], multichannel montages incorporating lateral occipital electrodes with full- and half-field stimulation may have value in detection of potential chiasmal and post-chiasmal dysfunction in future paediatric MOGAD cohorts [46, 47].

In conclusion, pRNFL thickness measured by OCT, particularly in the global, temporal, and PMB sectors, showed strong discriminatory capacity between paediatric MOGAD patients and healthy controls. In our cohort, these structural changes were also detectable in eyes without prior ON. The findings highlight the potential role of OCT as a non-invasive tool to support diagnosis and long-term monitoring in paediatric MOGAD, including in clinically silent or early disease stages. Future multicentre studies with larger cohorts and longitudinal follow-up may help confirm these findings and further characterise functional and structural changes over time.

## Data Availability

Anonymised data are available from the corresponding author upon reasonable request.

## Abbreviations

CRT: Central retinal thickness
HC: Healthy controls
HCVA: High-contrast visual acuity
LCVA: Low-contrast visual acuity
ON + /ON–: Eyes with prior/without prior optic neuritis
PMB: Papillomacular bundle
pRNFL: Peripapillary retinal nerve fibre layer
VEP: Visual evoked potential
YI: Youden index

## References

[1] Cacciaguerra L, Flanagan EP (2024) Updates in NMOSD and MOGAD diagnosis and treatment: a tale of two central nervous system autoimmune inflammatory disorders. Neurol Clin 42(1):77–114. 10.1016/j.ncl.2023.06.009 PMC10658081 37980124

[2] Marignier R, Hacohen Y, Cobo-Calvo A, Pröbstel AK, Aktas O, Alexopoulos H et al (2021) Myelin-oligodendrocyte glycoprotein antibody-associated disease. Lancet Neurol 20(9):762–772. 10.1016/s1474-4422(21)00218-0 34418402

[3] Jeyakumar N, Lerch M, Dale RC, Ramanathan S (2024) MOG antibody-associated optic neuritis. Eye (Lond) 38(12):2289–2301. 10.1038/s41433-024-03108-y PMC11306565 38783085

[4] Hor JY, Fujihara K (2023) Epidemiology of myelin oligodendrocyte glycoprotein antibody-associated disease: a review of prevalence and incidence worldwide. Front Neurol 14:1260358. 10.3389/fneur.2023.1260358 PMC10542411 37789888

[5] Al-Ani A, Chen JJ, Costello F (2023) Myelin oligodendrocyte glycoprotein antibody-associated disease (MOGAD): current understanding and challenges. J Neurol 270(8):4132–4150. 10.1007/s00415-023-11737-8 PMC10165591 37154894

[6] Santoro JD, Beukelman T, Hemingway C, Hokkanen SRK, Tennigkeit F, Chitnis T (2023) Attack phenotypes and disease course in pediatric MOGAD. Ann Clin Transl Neurol 10(5):672–685. 10.1002/acn3.51759 PMC10187731 37000895

[7] Havla J, Pakeerathan T, Schwake C, Bennett JL, Kleiter I, Felipe-Rucián A et al (2021) Age-dependent favorable visual recovery despite significant retinal atrophy in pediatric MOGAD: how much retina do you really need to see well? J Neuroinflammation 18(1):121. 10.1186/s12974-021-02160-9 PMC8164737 34051804

[8] Bruijstens AL, Breu M, Wendel EM, Wassmer E, Lim M, Neuteboom RF et al (2020) EU paediatric MOG consortium consensus: Part 4 - Outcome of paediatric myelin oligodendrocyte glycoprotein antibody-associated disorders. Eur J Paediatr Neurol 29:32–40. 10.1016/j.ejpn.2020.10.007 33183945

[9] Wendel EM, Baumann M, Barisic N, Blaschek A, de Coelho Oliveira Koch E, Della Marina A et al (2020) High association of MOG-IgG antibodies in children with bilateral optic neuritis. Eur J Paediatr Neurol 27:86–93. 10.1016/j.ejpn.2020.04.002 32327391

[10] Narayan RN, McCreary M, Conger D, Wang C, Greenberg BM (2019) Unique characteristics of optical coherence tomography (OCT) results and visual acuity testing in myelin oligodendrocyte glycoprotein (MOG) antibody positive pediatric patients. Mult Scler Relat Disord 28:86–90. 10.1016/j.msard.2018.11.026 30576847

[11] Corbali O, Chitnis T (2023) Pathophysiology of myelin oligodendrocyte glycoprotein antibody disease. Front Neurol 14:1137998. 10.3389/fneur.2023.1137998 PMC10011114 36925938

[12] Wendel EM, Thonke HS, Bertolini A, Baumann M, Blaschek A, Merkenschlager A et al (2022) Temporal dynamics of MOG antibodies in children with acquired demyelinating syndrome. Neurol Neuroimmunol Neuroinflamm. 10.1212/nxi.0000000000200035 PMC9562044 36229191

[13] Cobo-Calvo A, Ruiz A, Rollot F, Arrambide G, Deschamps R, Maillart E et al (2021) Clinical features and risk of relapse in children and adults with myelin oligodendrocyte glycoprotein antibody-associated disease. Ann Neurol 89(1):30–41. 10.1002/ana.25909 32959427

[14] Lin TY, Asseyer S, Buenrostro GS, Feldmann K, Hamann S, Paul F et al (2022) Neuro-ophthalmological presentation of optic neuritis in myelin oligodendrocyte glycoprotein antibody-associated disease. Klin Monatsbl Augenheilkd 239(11):1305–1314. 10.1055/a-1928-5117 35995144

[15] Bruijstens AL, Lechner C, Flet-Berliac L, Deiva K, Neuteboom RF, Hemingway C et al (2020) E.U. paediatric MOG consortium consensus: Part 1 - Classification of clinical phenotypes of paediatric myelin oligodendrocyte glycoprotein antibody-associated disorders. Eur J Paediatr Neurol 29:2–13. 10.1016/j.ejpn.2020.10.006 33162302

[16] Banwell B, Bennett JL, Marignier R, Kim HJ, Brilot F, Flanagan EP et al (2023) Diagnosis of myelin oligodendrocyte glycoprotein antibody-associated disease: international MOGAD panel proposed criteria. Lancet Neurol 22(3):268–282. 10.1016/s1474-4422(22)00431-8 36706773

[17] Trewin BP, Brilot F, Reddel SW, Dale RC, Ramanathan S (2025) MOGAD: a comprehensive review of clinicoradiological features, therapy and outcomes in 4699 patients globally. Autoimmun Rev 24(1):103693. 10.1016/j.autrev.2024.103693 39577549

[18] Eyre M, Hameed A, Wright S, Brownlee W, Ciccarelli O, Bowman R et al (2018) Retinal nerve fibre layer thinning is associated with worse visual outcome after optic neuritis in children with a relapsing demyelinating syndrome. Dev Med Child Neurol 60(12):1244–1250. 10.1111/dmcn.13757 29637998

[19] Jonzzon S, Suleiman L, Yousef A, Young B, Hart J, Peschl P et al (2020) Clinical features and outcomes of pediatric monophasic and recurrent idiopathic optic neuritis. J Child Neurol 35(1):77–83. 10.1177/0883073819877334 PMC7018758 31566057

[20] Chen JJ, Sotirchos ES, Henderson AD, Vasileiou ES, Flanagan EP, Bhatti MT et al (2022) OCT retinal nerve fiber layer thickness differentiates acute optic neuritis from MOG antibody-associated disease and Multiple Sclerosis: RNFL thickening in acute optic neuritis from MOGAD vs MS. Mult Scler Relat Disord 58:103525. 10.1016/j.msard.2022.103525 PMC8882134 35038647

[21] Marmoy OR, Viswanathan S (2021) Clinical electrophysiology of the optic nerve and retinal ganglion cells. Eye (Lond) 35(9):2386–2405. 10.1038/s41433-021-01614-x PMC8377055 34117382

[22] Odom JV, Bach M, Brigell M, Holder GE, McCulloch DL, Mizota A et al (2016) ISCEV standard for clinical visual evoked potentials: (2016 update). Doc Ophthalmol 133(1):1–9. 10.1007/s10633-016-9553-y 27443562

[23] Janáky M, Jánossy Á, Horváth G, Benedek G, Braunitzer G (2017) VEP and PERG in patients with multiple sclerosis, with and without a history of optic neuritis. Doc Ophthalmol 134(3):185–193. 10.1007/s10633-017-9589-7 28421377

[24] Tekavcic-Pompe M, Stirn-Kranjc B, Brecelj J (2003) Optic neuritis in children--clinical and electrophysiological follow-up. Doc Ophthalmol 107(3):261–270. 10.1023/b:doop.0000005335.28820.05 14711158

[25] Petzold A, de Boer JF, Schippling S, Vermersch P, Kardon R, Green A et al (2010) Optical coherence tomography in multiple sclerosis: a systematic review and meta-analysis. Lancet Neurol 9(9):921–932. 10.1016/s1474-4422(10)70168-x 20723847

[26] Schippling S, Balk LJ, Costello F, Albrecht P, Balcer L, Calabresi PA et al (2015) Quality control for retinal OCT in multiple sclerosis: validation of the OSCAR-IB criteria. Mult Scler 21(2):163–170. 10.1177/1352458514538110 24948688

[27] Oertel FC, Sotirchos ES, Zimmermann HG, Motamedi S, Specovius S, Asseyer ES et al (2022) Longitudinal retinal changes in MOGAD. Ann Neurol 92(3):476–485. 10.1002/ana.26440 35703428

[28] Shute RH, Westall CA (2000) Use of the Mollon-Reffin minimalist color vision test with young children. J Am Assoc Pediatr Ophthalmol Strabismus 4(6):366–372. 10.1067/mpa.2000.110335 11124673

[29] Hanley JA, McNeil BJ (1982) The meaning and use of the area under a receiver operating characteristic (ROC) curve. Radiology 143(1):29–36. 10.1148/radiology.143.1.7063747 7063747

[30] Hosmer DW, Lemeshow S (2000) Applied Logistic Regression, 2nd edn. John Wiley & Sons, New York

[31] Volpe G, Jurkute N, Girafa G, Zimmermann HG, Motamedi S, Bereuter C et al (2024) Diagnostic value of inter-eye difference metrics on OCT for myelin oligodendrocyte glycoprotein antibody-associated optic neuritis. Neurol Neuroimmunol Neuroinflamm 11(6):e200291. 10.1212/nxi.0000000000200291 PMC11379125 39231384

[32] Sotirchos ES, Filippatou A, Fitzgerald KC, Salama S, Pardo S, Wang J et al (2020) Aquaporin-4 IgG seropositivity is associated with worse visual outcomes after optic neuritis than MOG-IgG seropositivity and multiple sclerosis, independent of macular ganglion cell layer thinning. Mult Scler 26(11):1360–1371. 10.1177/1352458519864928 PMC6992495 31364464

[33] Yano C, Matsuura E, Nakamura T, Sonoda A, Shigehisa A, Ando M et al (2025) Visual evoked potential in myelin oligodendrocyte glycoprotein antibody-associated disease. Mult Scler Relat Disord 98:106408. 10.1016/j.msard.2025.106408 40174439

[34] Paul F, Calabresi PA, Barkhof F, Green AJ, Kardon R, Sastre-Garriga J et al (2021) Optical coherence tomography in multiple sclerosis: a 3-year prospective multicenter study. Ann Clin Transl Neurol 8(12):2235–2251. 10.1002/acn3.51473 PMC8670323 34792863

[35] Balk LJ, Steenwijk MD, Tewarie P, Daams M, Killestein J, Wattjes MP et al (2015) Bidirectional trans-synaptic axonal degeneration in the visual pathway in multiple sclerosis. J Neurol Neurosurg Psychiatry 86(4):419–424. 10.1136/jnnp-2014-308189 24973342

[36] Wicki CA, Manogaran P, Simic T, Hanson JVM, Schippling S (2020) Bilateral retinal pathology following a first-ever clinical episode of autoimmune optic neuritis. Neurol Neuroimmunol Neuroinflamm. 10.1212/nxi.0000000000000671 PMC7051214 31969471

[37] Motamedi S, Oertel FC, Yadav SK, Kadas EM, Weise M, Havla J et al (2020) Altered fovea in AQP4-IgG-seropositive neuromyelitis optica spectrum disorders. Neurol Neuroimmunol Neuroinflamm. 10.1212/nxi.0000000000000805 PMC7413713 32576604

[38] Gigengack NK, Oertel FC, Motamedi S, Bereuter C, Duchow A, Rust R et al (2022) Structure-function correlates of vision loss in neuromyelitis optica spectrum disorders. Sci Rep 12(1):17545. 10.1038/s41598-022-19848-4 PMC9585067 36266394

[39] Vabanesi M, Pisa M, Guerrieri S, Moiola L, Radaelli M, Medaglini S et al (2019) In vivo structural and functional assessment of optic nerve damage in neuromyelitis optica spectrum disorders and multiple sclerosis. Sci Rep 9(1):10371. 10.1038/s41598-019-46251-3 PMC6637174 31316082

[40] Geraldes R, Arrambide G, Banwell B, Rovira À, Cortese R, Lassmann H et al (2024) The influence of MOGAD on diagnosis of multiple sclerosis using MRI. Nat Rev Neurol 20(10):620–635. 10.1038/s41582-024-01005-2 39227463

[41] Fadda G, Alves CA, O’Mahony J, Castro DA, Yeh EA, Marrie RA et al (2021) Comparison of spinal cord magnetic resonance imaging features among children with acquired demyelinating syndromes. JAMA Netw Open 4(10):e2128871. 10.1001/jamanetworkopen.2021.28871 PMC8515204 34643718

[42] Camera V, Holm-Mercer L, Ali AAH, Messina S, Horvat T, Kuker W et al (2021) Frequency of new silent MRI lesions in myelin oligodendrocyte glycoprotein antibody disease and aquaporin-4 antibody neuromyelitis optica spectrum disorder. JAMA Netw Open 4(12):e2137833. 10.1001/jamanetworkopen.2021.37833 PMC8655599 34878547

[43] Obuchowski NA (2003) Receiver operating characteristic curves and their use in radiology. Radiology 229(1):3–8. 10.1148/radiol.2291010898 14519861

[44] Leite MT, Rao HL, Weinreb RN, Zangwill LM, Bowd C, Sample PA et al (2011) Agreement among spectral-domain optical coherence tomography instruments for assessing retinal nerve fiber layer thickness. Am J Ophthalmol 151(1):85-92.e1. 10.1016/j.ajo.2010.06.041 20970108

[45] Šuštar Habjan M, Bach M, van Genderen MM, Li S, Mizota A, Nilsson J et al (2025) ISCEV standard for clinical visual evoked potentials (2025 update). Doc Ophthalmol 151(2):97–112. 10.1007/s10633-025-10042-1 PMC12436483 40839165

[46] Thompson DA, Marmoy OR, Cowe J, Handley SE (2025) Multi-channel pattern veps with full and half field stimulation: methods of interpretation and diagnostic evaluation. Doc Ophthalmol 150(2):87–95. 10.1007/s10633-025-10012-7 PMC11991936 40128423

[47] Handley SE, Šuštar M, Tekavčič Pompe M (2021) What can visual electrophysiology tell about possible visual-field defects in paediatric patients. Eye (Lond) 35(9):2354–2373. 10.1038/s41433-021-01680-1 PMC8377065 34272512

